# Abstract of the Discussion in the Pathological Society, London, June and July, 1873, on the Anatomical Relations of Pulmonary Phthisis to Tubercle, with Remarks

**Published:** 1873-12

**Authors:** Z. Collins McElroy

**Affiliations:** Zanesville, Ohio


ARTICLE III. — *Abstract of the Discussion in The Pathological Society, London, June and July,* 1873, *on the Anatomical Relations of Pulmonary Phthisis to Tubercle, with Remarks.* Reported to the Zanesville Academy of Medicine, Zanesville, O., Oct. 21st, 1873, by Z. COLLINS MCELROY, M.D., of Zanesville, Ohio.

During the months of June and July, present year, 1873, a discussion took place in "The Pathological Society" of London, "On the Anatomical Relations of Pulmonary Phthisis to Tubercle," which was, in many respects, one of the most remarkable ever had in a medical body, for the acknowledged giants of the profession of that city were the contestants.

The debate was characterized by the almost extreme courtesy of the speakers to each other; entertaining, as they did, very diverse opinions on many points, which none seemed afraid to bring forward. Then, again, there was an equally remarkable agreement as to the more obvious, as well as minute and technical points of pathological histology, after death from so-called phthisis. These were illustrated by microscopical preparations, and drawings, enlarged images of which were shown on screens. On these there was a substantial agreement, but there it ended, for the interpretations, lessons, inferences, or deductions, drawn from these changed conditions of structure, varied very widely. And the general results of the discussion were both positive and negative, the latter preponderating.

Perhaps one of the most noticeable positive results was the clean sweep made of current pathological notions, opinions, or speculative beliefs, of the intimate relationship between tubercle and phthisis, as cause and effect. So far from this being the case, it was made evident during the discussion, that the very reverse is probably correct, viz., that tubercle, so called, was a result, or at least only a concomitant, not alone of the condition called phthisis, but of pneumonia, scrofula, and divers other conditions.

Gentlemen in the profession who have posted up in their minds as a finality, that phthisis, so called—consumption, popularly—depends on tubercles in the lungs, are in a Rip Van Winkle sleep, and if they ever wake up, will find their pathology out of fashion entirely.

Dr. Wilson Fox, who opened the debate, made a statement of the anatomical condition of the lungs of children dying from typically acute phthisis; and describes them as follows:

"Semi-transparent granulations; white granulations, neither absolutely opaque, nor absolutely semi-transparent, with a certain degree of firmness. Granulations like the semi-transparent, firm, and more or less caseous in their center. Yellow, soft granulations, somewhat prominent, easily crushed, but not easily removed from lung tissue, varying in size from a poppy to a mustard seed, and more rarely a split pea. Then, distinct caseous granulations, sometimes with, and sometimes without, a semi-transparent zone surrounding them. Groups semi-transparent, or opaque granulations, one, two, three, or more in number, reaching the size of the split pea, or bean. Indurated granulations surrounded by a zone of pigment. Tracts of indefinite extent, one, two or more inches in diameter; irregular in outline, granular on section, which sometimes pass insensibly into so-called gray infiltration. Cavities, from an infinitesimal speck to that of hazel nuts, or larger. Sometimes granulations softening into cavities, either the softer, white, or yellow. The semi-transparent granulations, *per se*, as far as I have seen them, are not found softening into a cavity, without some intermediate change. Tracts of gray, semi-transparent, gelatinous in appearance, what we know as either gray pneumonia, or gelatinous pneumonia, or gelatinous gray infiltration; and red pneumonia, bronchitis injection, or punctiform extravasation; and dilatation of bronchi, and some other appearances, having little to do with the subject under consideration.

"The point I wish to insist on is, that the gray granulation—typical tubercle of Virchow—does not exist alone in the majority of cases of acute tuberculosis of the lungs of children, but is almost always found with other forms of change, which, in a large majority of cases, so predominate that the gray granulation is the rarer appearance, and is even difficult to find.

"By gray granulation I allude to a body composed of minute cells about the size of a white blood corpuscle, or smaller in older forms, separated by a delicate net work, and may be taken as the typical structure of tuberculosis. There are often much larger cells, sometimes the $\frac{1}{200}$ or $\frac{1}{300}$ of an inch in diameter, with many nuclei. I have never been able to isolate them. Those characteristics of tubercle in the lung are these small cells, with a nucleus, often nearly filling the cell wall.

"The point I wish to lay before the society is, that all these changes—caseous infiltration, large pneumonic product; mingling of pneumatic products with reticular growth; induration of reticular growth; infiltration of that reticular growth throughout large parts of the lung, which then acquire a more or less caseous appearance--all concur in the most typical forms of acute tuberculosis.

"I venture to lay down these propositions as corollaries to these points:

"1st. Speaking in the abstract, tubercle may occur without inflammation in the elements of the tissue in which it is developed.

"2nd. That tubercle is found in addition to inflammation of the tissue in which it occurs.

"3rd. That in the liver and kidney in certain blood states, you may have tubercular growth, as part of the signs of the acute disease, or constitutional affection, in which no inflammation of the part in which it occurs can be detected.

"4th. That in mucous and serous membranes, or tissues, the mixture of inflammation and tubercle is constant.

"5th. That in the lung the implication of the cell wall (air cell) is the most constant and typical lesion of phthisis. Excluding ulcerative bronchitis, ulceration after pneumonia, indurated pneumonia, lesions from dust, cancer, and syphilis. I have, during my life, only found three cases of phthisis which did not present the gray granulation, or soft granulation, or such caseous change, or infiltration such as I have described.

"Chronic phthisis is distinguished from acute by the slowness of induration in these growths, and not by their quick or acute destruction.

"I have given no definition of tubercle, and am not going to do so; but so far as we know at present of tubercle, it is a lymphatic overgrowth produced by irritation. Lymphatics have been traced in sufficient numbers in the cell wall (air cell) to allow us to believe that lymphatic tissue must play an important part in the changes of lung tissue."

Dr. Moxon approved of the statements of Dr. Fox, and summarized them as follows:

1st. That miliary tuberculosis of the lungs has neither the anatomical nor histological constancy, or peculiarity, commonly ascribed to it.

2nd. That miliary tuberculosis, in short, exhibits all the products found in active chronic phthisis.

3rd. That all the other products, constituting caseous pneumonia under various forms, are essentially of the same histological structure, which Dr. Fox thinks is characteristic.

4th. That the other characters of chronic phthisis are fairly traceable to time.

5th. That he, Dr. Fox, asserts the identity of all phthisis, based on this peculiar microscopic matter.

Dr. Moxon did not think that the basis of a definition of phthisis should rest on purely histological grounds. There was nothing specific about it. If inflammation was set up in a person, he might be said to be on the high road to tubercle. Did not think due attention had been given to the developmental changes in tubercle. Thus, the life of a tubercle was inside of three months, so that in five or six years — the time allotted to so-called fibroid phthisis — many generations of tubercle must have come and gone, doing their natural work in destroying, first, their matrix, and then themselves, leaving behind them the scar of fibre in which the nature of our bodies always enshrouded the traces of such mischief.

Of tubercle, since we can define its cause, it is not specific. Neimeyer's view, that tubercle is a development of inflammation, is essentially weak — not sustained by facts.

Dr. Cayley thought that if tubercle was to be regarded as a lymphatic overgrowth caused by irritation, then tubercle cannot be distinguished, since the so-called adenoid tissue, to which the term lymphatic overgrowth by irritation was applied, occurs in parts of the body free from any kind of irritation. It is found in the margin of hard chancre, in a soft node, in early stages of cirrhosis of the liver, and can be produced in lung tissue by a great variety of causes, as grindey dust, chronic pneumonia, pleurisy, etc. Thought the term tubercle should be restricted to the gray granulations.

Dr. Beale agreed as to the descriptions of Dr. Fox, but differed in several important points of interpretation. Did not think the tubercle corpuscle had anything to do with lymph corpuscle, or adenoid tissue. Thinks that "irritation" is too loose a term. Virchow's doctrine of irritation would not stand scrutiny, for it leads to the ridiculous conclusion that minute particles of protoplasm, bioplasm, or whatever else they may be called, have the power of sympathizing with other particles. The term irritation must be looked upon as the Abracadabra of pathology.

"It seems to me that though it may be impossible for any one to say that this or that is a tubercle corpuscle, we must assent to the doctrine that it is a special thing. It would be as impossible to distinguish any one of these compound masses of different forms of living matter, as to distinguish the corpuscles of the embryo of one animal from the other, if they were mixed together. Under different circumstances, no doubt, the tubercle corpuscle will give rise to a differently formed material, fibrous, soft, glistening, or a matter which at last may degenerate into that which we know as caseous substance."

Dr. Bastian said the notion that cheesy degeneration, or caseation, constituted the essence of tubercle, must be thrown aside, as one which a larger experience could in no way sanction. The characteristics relied upon by Laennec, and others, had been shown to be almost wholly valueless as characteristics of any special and peculiar something, which it had become the fashion to call tubercle. Inasmuch as such products, both in their incipient and advanced stages, were specially abundant in the lungs of persons dying from phthisis, this affection became one of which tubercle could no longer be considered the pathological essence. It was now known that epithelial impactions, and overgrowths in the bronchial tubes, and fibroid infiltrations of a chronic inflammatory character or nature, were, in the main, the special tissue changes which subsequently gave rise to the various lesions of chronic phthisis.

Objects to conclusions founded on experiments on rodent animals in the artificial production of tubercle. Believes them to be so peculiar as to render any conclusions hastily formed from experiments on them, unsafe to apply to human pathology altogether. And is impressed with the altogether gratuitous and unnecessary difficulty besetting the paths of teachers who endeavor to explain to students what is and what is not tubercle. To this conclusion he has come slowly and deliberately, after years of experience as a teacher.

Dr. C. I. B. Williams cannot but think that this debate on tubercle has been more about words than things. A great many things have been shown, and there is ample proof of much diligent labor and careful observation, more, apparently, with the object of finding out what these things shall be called, than to find out their real nature.

"The term growth, or growths, is applicable to tubercle only in the most limited sense. Except at their first inception they do not grow as other growths and tumors do. They harden by the increasing number and consistence of their corpuscles. And this induration, by depriving them of pabulum from the blood, leads to their ulterior decay, either by caseation or dwindling. As growths they are insignificant and abortive, and their chief characteristic is early decay. This is the foundation of their consumptive character, tending to the destruction of the tissues, and waste of the body. That miliary tubercles are essentially modifications of lymphatic glandular tissue, is, I think, fairly proved by recent observations, confirming the opinions of Portal, Broussais, Abercromby, and others. The similarity in scrofulous disease in lymphatic glands, and tuberculous disease in the lungs, and their succession in the same individuals and families, have been generally accepted as strong evidence in favor of their identity; and rendered most probable the views of Portal and Broussais, founded on anatomical as well as clinical observations, that miliary tubercles have their seat in the lymphatic textures. Virchow's declaration that tubercle resembles lymphatic or adenoid tissue, together with recent microscopic observations, have produced a general concurrence of opinion as to their resemblance, if not identity. I do not concur in Dr. Fox's declaration, that tubercles are lymphatic overgrowths; nor do I believe the lymphatic system is at all necessary to the production of other tuberculous formations not granular. There is an intimate connection between the form of miliary tubercle and some elementary parts of lung structure.

"The corpuscles of miliary tubercle are lymphatic, developed by irritation in adenoid tissue; while the corpuscles of inflammatory irritation are the pale blood particles, migrating, and forming the corpuscular exudation matter of scrofulous and other low types of inflammation. And since lymph corpuscles are identical in all their characters with the colorless corpuscles of the blood, so we find the same resemblance in appearance, and the same unity in nature, in the multiplied corpuscles of diseased lymph in miliary tubercle and those of inflammatory exudations in scrofulous subjects. In this there is my key to the two-fold seat and origin of tubercle, or rather of consumptive disease:

"1st. Lymphatic; miliary; infective; scattered.

"2nd. Inflammatory; diffused; local.

And thus we get explained the identity, and yet the difference, of all the chief elements of consumptive disease—granular and diffused—differing in form and seat, but alike in their corpuscular composition and proneness to decay.

"In ordinary nutrition, and even in hypertrophy, the growth of the tissues may be affected by the activity and proliferation of their proper cell germs, or bioplasm. But in inflammation, and similar states of vascular excitement, there is an overflow of plasma through the coats of the vessels, with nascent materials, ready to form without the aid of any cells or germinal matter from old tissue. The result is the coagulable lymph of Hunter; the fibrinous and croupous of Rokitansky; the fibrinous and corpuscular of Paget; the plastic and cacoplastic of my own; thus accounting for the relations of tubercle or phthisis to inflammation more satisfactorily than the maze of growths in which the northern Germans mystify the subject. The more fibrinous and less cacoplastic exudation becomes organized into a tough contracting tissue called fibroid, and other like chronic consolidations. The more corpuscular and aplastic degenerate into cheesy matter, to soften and decay. constituting yellow tubercle, and similar results of scrofulous pneumonia. And these phthinoplasms, as I call them, whether they wither and dwindle, like fibroid, or caseate and decay, as tubercles, and corpuscular indurations, represent different degrees of the same consuming disease, which brings the flesh-forming, and life-giving materials of our body to premature decay."

Dr. Green thought, on the whole, it were better to drop the term tubercle, since its retention tends to cause confusion among pathologists, from the utter impossibilty of saying that this is or is not tubercle.

Dr. Crisp thought that the study of such a question as the relations of tubercle to phthisis ought not to be confined to the human body, but extend to the whole range of inferior animals, determining, by experiment, in what kind of organization each histological structure was first met with, and how it differed from that in man. In this ascending line of investigation the discrepancies and differences now existing might be satisfactorily explained and harmonized.

Dr. Pollock reviewed at length the history of tubercle, from Laennec down to the present time; and concluded, that while there was typical pathological unity in phthisis and tubercle, the disease called phthisis was the manifestation of various morbid agencies, lymphoid, inflammatory, and degenerative, which, in their various evolutions, constituted the several varieties of the clinical affection.

Dr. Wilson Fox closed the discussion very lengthily, going all over the ground again; endeavoring to answer objections, and reconcile facts and appearances, but admitted that the search for specific or definite pathology for a specific or definite disease, or diseases, had been a failure; and that the anatomical and pathological relations of tubercle to phthisis were still chaos. He wouldnot, however, in consequence, drop the term tubercle, nor for the present cast aside names for diseases in our literature. He had began the study of phthisis, impressed with the belief that there were a number of specifically different diseases grouped under the general name phthisis, but his investigations had long since convinced him to the contrary.

The foregoing abstract of the debate, or discussion, was taken from the printed reports as they appeared in the American reprint of *The Lancet*, for July and August, and are mostly in the very words of the several speakers, as published. Sentences were selected in which they seemed to approach definiteness in their statements. It has been made with care, and though more or less broken and detached—scrappy, as it were—and lacking the fullness of the thirty closely printed pages reported in *The Lancet*, yet, I think, fairly conveys the conclusions of the several speakers.

In commenting on the discussion, the first thing to be noticed as striking and peculiar, is, that a discussion on such a restricted topic as the anatomical relations of phthisis to tubercle, occupying so much of the time of men who probably have little to waste, should have taken place at all. Nor need it be an occasion for surprise that no positive results were reached, since it must have been known to the speakers themselves, as well before as after the discussion, that there were none to be reached.

Several of the speakers apparently chafed under the restraints imposed on them by the nomenclature in use by the profession to designate appearances only, but not the facts behind them.

An effort to get the term tubercle dropped out of use was made, but it was unsuccessful. Another term in still more frequent use—irritation—was severely handled. But the weight of "authority" was against disturbing the technology now in use, and the matter was dropped. And yet that is the most urgent need of medicine now. Many of the terms used in medicine have descended to us from the remotest antiquity unchanged in significance. Their definitions cannot be changed, and the only alternative is an entirely new nomenclature, to correspond with the facts which underlie appearances. Entire changes in nomenclature have been made in other departments of knowledge, a notable example of which is seen in chemistry. What was ferri sulphus, when I was a student of chemistry, is now feric sulphide. We who now write for "bromide of potassa," or, in the language of the Pharmacopœia, "potassii bromidum," will find it "potassic bromide" in our new works on chemistry. Of course this change has not been made without opposition. But it is being gradually adopted, and at the end of another generation will probably be in universal use.

Now what has been done in chemistry, and is now doing in chemistry, can be done in medicine, though perhaps more slowly. But as *The Philadelphia Medical Times* puts it. "'Nestor' says, leave innovations alone, or you will lose your bread and butter." And so bread and butter may, for many generations, prevent the necessary modifications in medical nomenclature, to the end that it may take rank with other sciences in exactness.

There was not, in all probability, a solitary Fellow of the Pathological Society of London, at that time, who did not know that life—all life, animal and vegetable—on our earth, has but three fundamental factors, viz., materials, forms of structure, and motion in materials of structure; or, in other words, materials, force, and forms of organic structure; and that they had no power either to create, or annihilate, either materials or force; and, except through natural channels, or modes, they could not create or make or combine materials into an organic form of structure, capable of performing a function. And that, as physicians, their sole power laid in a partial control of force, or motion in materials of structure. That to study, or discuss properly the anatomical relations of any given symptoms, or phenomena, before death, with the conditions, or states of structure, or materials of structure, after death, the whole of the conditions of life—evolution, growth, maturity, decline, and dissolution, or, in other words, histogenesis and histolysis of the tissues—must be brought under consideration. Yet, guided largely by nomenclature, they discussed a narrow, and, in fact, not a discussable question.

As pertinent to the question, and a necessary preliminary to its discussion, an approximate or definite settlement must be arrived at on the following points: What relation does the body of man bear to the materials and forces of nature about him? How do the materials he uses as food, the water he drinks, the atmosphere he inhales, combine to form living flesh, capable of performing a function, or functions, chief among which is repair from new material as momentarily wasted; and multiplication and perpetuation in gross of his body? Is it by the ordinary forces of nature, "stored up" in the food he eats, and made available in his body, by "modes" during its complex chemical changes there? Or, is it by some special force not manifested elsewhere in nature than in living bodies? Is it by chemical means, *i. e.*, by reaction between elementary atoms or molecules of matter; or by mechanical means, as in building houses, bridges, or ships? What are the means by which the structures are maintained? Is it by chance, or accident, or does it take place in obedience to law and order? Has what we call force anything to do with the metamorphosis of food into living flesh? Are the materials so varied in form and color found in the lungs after death from so-called phthisis, matter which has failed to reach natural structural condition? Or, is it matter on its downward career from its condition in natural living structure? What are their ultimate constituents, and in what respect do they differ from the food eaten during life? Are they from food at all? In what respect do they differ from the ordinary condition of similar elements elsewhere in nature? Are their existing conditions—chemical and physical appearances—due to a deficiency or excess of force, or misdirected energy in either the ascending or descending scales of organization? Are they due to conditions or causes internal or external to the body? If so, what, and why? How much is certainly known, and how much extremely probable, in regard to it?

These, and many more like questions, must be answered and settled, or approximately settled, before any intelligent inquiry can be made into the relations—anatomical relations—of states of the living body, with post mortem appearances.

These questions are not asked because they are supposed to be posers, as difficult questions were called in my school-boy days; or because they cannot all be answered either certainly, or approximately, with almost certainty. For there was not one of the speakers in the debate in the Clinical Society who could not have done so then, and cannot do so now. The truth is, the question they discussed was not such an one as its learned fellows ought to have discussed, for the results obtained were not a compensation for the work performed. No such narrow topic can ever be discussed properly, because the factors to it are at best only hypothetical, or traditional, founded only on external appearances. We all talk, learned and unlettered, of sunrise and sunset, yet most of us know that the sun neither rises nor sets, as the words would imply, as ordinarily defined. Thus, the conditions of living bodies designated phthisis, are so called on hypothetical considerations only. The peculiar chemical state, and physical appearances of materials, found in the lungs, and elsewhere, after death, are called tubercle, on hypothetical grounds only; and conclusions or deductions drawn from such hypothetical premises could not and did not disclose any certainties, as this debate has so strikingly and clearly demonstrated.

Two years since, a large part of a populous city in the State of Illinois was laid waste by fire,—who does not remember the Chicago fire? Nothing of consequence was saved from the burnt district; all property, and some life, shared a common fate in the conflagration, so rapid were the chemical changes in the material there.

Now, Chicago before the fire, so called, was composed of organized and unorganized material, having forms of structure to adapt it to the performance of the functions of the traffic and life concentrated there.

And Chicago before the fire, and a human body before death, differ not so much in material, or in forms of structure to perform special functions, as in this fact, that while both perform function at the expense of structural forms, Chicago, as a city, possessed no inherent capacity for repairing daily waste from new material, while living bodies have that inherent capacity, but not to an unlimited extent, else living bodies would be immortal. The conflagration in Chicago destroyed no material, it only changed its forms of structure, as an incident to the changes in the chemical condition of its material. And no force was lost or destroyed; but the force stored up in its materials was suddenly liberated, and found its correlation in mechanical motion in the surrounding atmosphere, and in the new chemical conditions of its material, which then presented other and widely different physical appearances.

Without repair of its structures year by year, as they were wasted in the performance of function, time would have completed the work of the destruction of Chicago as completely and effectually as the conflagration. But the *post ignum* appearances and condition of its matter would differ widely from their *post mortem* appearances after natural decay.

And so of living bodies — human bodies — their capacity for self-reproduction is limited in time. Little by little the forms of structure are lost, as the post mortem appearances of the octogenarian abundantly demonstrate. Time,* and wear and tear, complete their work here, just as it has done for cities as populous as Chicago was, in the past.

Life is terminated by various causes at all stages, suddenly, by missiles of war, accidents incident to civilization, so-called diseases, as from chemically complex matter introduced from without, capable of setting up higher rates of motion, and changing forms of

* We all speak of time as something absolute, or real. But this is only true in the same sense as we speak of the sun rising or setting. Time is the relative and ever changing relation of moving masses of matter to each other. There is no abstract time, *i. e.*, a something distinct from the relative position to each other of masses of matter, large or small. There are no two points on the earth's surface having the same apparent or real time, at any moment. The reality behind what is called time, is motion by matter.

structure, as the virus of serpents and insects; or the virus whose results are severally designated small pox, yellow fever, Asiatic cholera, etc. Here, post mortem appearances differ widely from those of the octogenarian, mainly wrought, as we say, by time, but, at all events, by causes operating slower than those giving rise to the states named.

Again, cities have been desolated by violence, other than fire, *i. e.*, mechanical motion in their material, as by explosions, incidents of war, earthquake, etc., more speedily even than was Chicago by chemical motion in its materials — fire, though the results are the same, viz, forms of structure, upon which their utility and value to human society depend, are broken up, and capacity for function lost. The physical appearances of their material would vary as the motion in it had been mechanical or chemical, and time occupied in effecting them; the force in both cases being the same, and differing only in mode and velocity.

Like many thousands of my fellow men, I have made a *post ignum* examination of the burnt district of Chicago. I found that all material capable of assuming gaseous or liquid conditions during the fire had disappeared; and in place of the *ante-ignum* Chicago, with its tide of life and traffic, there only remained the non-gaseous matter, forced into new and often curious combinations. The mass of iron in the Park — a memorial — weighing several tons, existed in some commercial establishment before the fire as kegs of nails.

My lesson from a *post ignum* examination of the burnt district of Chicago, was not to note and map the forms, appearances and colors its material presented. A new city had in part arisen, and was rising, from amongst the ruins or *debris* of the old, which had so suddenly passed away. The materials of the new city were partly old, and partly new, the new largely predominating. But it was still Chicago. Of this, more presently. A good many of the later years of my professional life have been devoted to the study of my own body, and naturally enough the question "how does the food I eat become part of my living flesh?" came up for solution. The hypothesis of a special vital force as the means, though accepted as solving the problem during so many centuries, was soon dropped out of consideration. Then I found it necessary, too, to similarly dispose of much of the physiology now currently accepted on the subject, before any progress was made in obtaining a solution satisfactory to my own mind. Then, collecting the facts of experiment and observation obtained by others, and adding to them facts and observations, together with some experiments, of my own, I started out *de novo* to track food to living flesh. And I now entered on the investigation without any preconceived ideas on the subject, other than those from which I had endeavored to divest myself. In prosecuting my inquiries I soon found myself in possession of a considerable mass of facts, which did not fit well together. Men of science point out the uselessness of mere collections of facts, as well as the mistake of confining investigations concerning their relations to each other within too narrow limits. They have shown, also, the importance of comparing the ascertained facts of one subject, with the facts of other and apparently little related subjects. In this way much useful information, and many instructive analogies, are obtained. It is from facts viewed in relation to known conditions that we can alone hope for useful additions to our knowledge.

I was not long in discovering that my own body presented too many complexities to allow a solution to be reached from its study, and it became evident that I must get the key among simpler conditions of life.

In the vegetable world I found the means for reproduction, *i. e.*, the force, and part of the material, stored up in what is called a seed. That to grow, or perform its function, a seed must be surrounded by certain conditions, as new material, moisture, atmosphere, sunlight, and a certain range of heat, that is, motion of matter, and protection from enemies. That when the seed was ripe, that is, means perfected for reproducing, multiplying and perfecting its kind, in the case of annuals, the parent plant was dead, or died soon after; and with perennials, coincident with the fall of foliage. That there was individuality in the vegetable realm of nature, as the animated. That to preserve identity, grafts, and shoots, or cuttings from the parent plant, must be used in multiplying any special kind for desirable fruits, or flowers, as apples, pears, grapes, roses, etc. That the seed reproduced its kind, but not with perfect identity, for each new plant from a seed had its own individuality.

In this scrutiny of the vegetable world, it did not escape my notice, as I have already stated, that the ripening of the seed—that is, storing up the force in the necessary material to form the structures of the new plant up to that point and time when it could appropriate new, and other material, not supplied, or stored up by the parent plant, to its further growth and evolution—was simultaneous with the decay of the parent plant.

Then, my observation had shown me that the old Chicago had stored up the means prior to its destruction, or death, by fire, not in material, but in its trade and commerce, to rebuild itself, partly from old, but mostly from new material, which were analogous precisely with the conditions for reproduction in the vegetable world.

With this information I returned to the human body, to inquire whether there was in it any like, or analogous, provision for converting food into living flesh, continuously, during the life of each individual. Would the law that the old stored up the force for its own reproduction from new material, apply to the human body? I concluded that it would, for the law seemed to be universal that the new must proceed from the old. To be applicable, then, to the human body, there must be an apparatus fitted by structure and distribution to separate the material in which the requisite force was stored up, from the general debris of the tissues, and restore it, or unite it, at a proper place and time, with new material, to the blood stream.

There is in the human body an elaborate and complicated apparatus, distributed to every part of it, to which no adequate function has, up to this time, been assigned by physiology, to wit, the lymphatic system. Economy of material, catching up that accidentally escaping from the blood vessels, was assigned as its chief function elsewhere than in the abdominal cavity. Here, though identical in histological structure, a different function was assigned, viz., to take up the products of digested food and convey them into the blood.

As such an arrangement, to wit, separate functions at different points, for identical structure, histologically, would be a violation of the laws prevailing elsewhere through organic life, accepted physiology in regard to the so-called lacteals and lymphatics was probably in error, and was, therefore, with the vital force set aside, and a uniform function attributed to the system throughout the body; the largely increased volume of material found in the lymphatics or lacteals, during active digestion, due to increased waste of structure as a consequence of the increased duty performing, for here, as elsewhere throughout nature, organized and inorganic, function is performed at the expense of structure and structural arrangement of material.

Accepted physiology in regard to the entire digestive process, so called, is probably in a very incomplete condition. Admitting, for the moment, that the function of the lymphatic system is as here pointed out—and it corresponds in dignity with its histological structure and distribution, while the functions now attributed to it are sadly lacking in that respect—it becomes possible to trace food through its various stages and processes to living flesh more intelligently than through present physiological conceptions of function of the several parts concerned.

The food eaten by human beings is by chemistry arranged into a few groups of dissimilar chemical arrangement of material, viz., fibrins, albumens, gelatin, starch, fat, sugar, gum, etc., etc., from animal sources and vegetable seeds, and in a few instances the bodies of vegetable plants, in which some woody fibre and insoluble matters enter.

Now, it seems to me the sole function of the stomach is to effect solution of these "proximate principles" of animal and vegetable structures or materials. This is accomplished through several agencies, as pepsin, acids, etc., but mainly by water. When solution is effected, it is passed directly into the venous blood stream,* and goes forward to the liver. Here, some not well understood chemical changes occur, prominent among which is the transformation of starch into glucose. But, whatever they are, the result is sent forward to the right heart on its way to the lungs.

The stream pouring into the right heart—blood pump, simply—would therefore be thus complex:—

* Physiology acknowledges that a part of the material introduced into the stomach as food, pursues this path. My conclusions are, that all follow in the same way; and so rapid is the process of repair of wasted structures from new material, that a tired man's meal is partly in living structure before it is finished. And it is so also in sickness; proper food is rapidly disposed of in the same way. In many conditions of sickness—and that called phthisis, among them—this capacity is lost, from failure of the function of the lymphatic system in part.

1st. Old material—venous blood, from the debris of the tissues of all parts of the body.

2nd. New material, from food introduced into the stomach.

3rd. Lymph, containing the material in which the wasting structures have stored up the force for their own reproduction from new material.

In the lungs this complex stream meets the gaseous atmosphere, changes, not now understood in detail, take place, a certain proportion of effete material being detached, and making its exodus from the body at the mouth and nostrils.

The stream returning to the left heart differs chemically, and in physical appearance very widely, from the ingoing stream—arterial blood—the material in which is stored up the force for the reproduction of the momentarily wasting structures as function is performed. From the left heart it is distributed to the capillaries, the actual seats of molecular waste and repair. It seems to me that this synthetical tracery of food to living flesh points to the lungs as the actual seat of what is known as digestion, if that means the bulk of the chemical changes in food to fit it to become living flesh.

In good health we know nothing of our lymphatic system. And in sickness it has been little studied, except when there is palpable and visible modifications of its glandular structures. Scrofula is the general term expressive of these more obvious changes and failures of structure, confessedly conditions not much under remedial control; for not alone is the lymphatic system in disorder, but all the other structures, depending, as it seems to me they do, for their forms of structure and capacity for function in the general economy of each living body, on the lymph.

Viewed thus, to discuss the question of "the anatomical relations of tubercle to phthisis" in nature and essence, was an error, and could not by any possibility lead to other than negative results.

Certain other great general principles have been reached in my life-long investigations, which I formulate as follows:

1st. The main purpose of all life, animal and vegetable, in the general economy of nature, is its own preservation, reproduction, and multiplication.

All other phenomena are incidents only of this greater purpose.

Flowers, in the vegetable world, are only incidents of the formation of the seed. Intellectual phenomena in animal life are, in like manner, only incidents of the capacity, exercised or not, of self-reproduction and preservation; are not essential, are added, in virtue of "free moral agency;" and the structures on which they depend, not being essential to self-preservation, perish with each possessor, or leave only, or transmit only, increased "possibilities" of reproduction by processes known as "educational."

2nd. That for each special function performed, simple or complex, there is behind it, and on which it depends, special molecular arrangement of material; and as this special arrangement of material is modified, wholly changed, or rate of motion in its material advanced or retarded, function is correspondingly modified or lost.

3rd. That function is always, and under all circumstances, performed at the expense of special molecular arrangement of material, or structure.

This is the basis of all "diagnosis" in practice, not entirely empirical. Nosology and nomenclature for changed functions are the barriers which prevent the speedy attainment of the highest skill in "diagnosis," by the rank and file of the profession. Far too much attention is now given to naming things, and too little to ascertaining the reality of things, in sickness.

4th. That function is, in like manner, the index, language, or expression of molecular arrangement of material, or structure, from which it proceeds.

The vast quantities of material annually passed through the commercial channels of Chicago, which ultimately become animal structures, do not perform functions of life until they have been arranged in molecular forms of structure. Corn, oats and hay will not transfer merchandise, produce, or propel street cars, or any other actual functions performed by the various animals daily driven through its streets, until transformed into animal tissues. These materials may be burned in furnaces, producing, as the single phenomena of their combustion, the motion of matter called heat, which may be utilized by correlation in mechanical motion, but cannot be made in this way to perform the complex functions of animals.

So, automaton imitations may be and are made to move, produce sound, and imitate other phenomena of life, or living things, but they lack both fitness and adaptability, and the capacity of self-perpetuation and reproduction from new material, of the realities of which they are merely outward resemblances.

5th. That new life always rises from among the *debris*, or decaying material of the old.

The physical appearances of material found in the lungs of persons dying from so-called phthisis, was a question of force, and not of the forms and colors of materials. In the condition thus designated, there is conspicuous failure, or waste of structure, without corresponding repair from new material, from, as I understand it, failure of the necessary modes of force. At the velocity of waste in what is called acute phthisis, the structures cannot, and do not, store up the force for their own reproduction from new material, as they decay in the performance of function. In chronic, so called, phthisis, the functions of the lymphatics probably fail first, in not making the necessary separation of the material from the general debris, in which is stored up the force for the reproduction of the wasting tissues. And as the force is increased, lessened or varied, so will the forms, colors and appearances of material vary, found on post mortem examinations; and be without constancy; as this debate of the Pathological Society so clearly demonstrated.

*Cui bono?* of all this, asks a veteran therapeutist, who never thinks of, or wants to know, anything else than that this or that drug, or medicine, or compound, is good for this or that disease, or class of so-called diseases.

Just this, I reply—the good that all truth does. The function I have assigned to the lymphatic system has much positive and more negative evidence in its support, besides harmonizing with all the facts of organic nature. Its tendency will be to render us all increasingly exact in our professional services to the sick. We can hardly fail to be wiser, and better fitted for our duties, the more intimately we become acquainted with the inner workings of our wonderful bodies.

